# Antimicrobial resistance patterns and prevalence of class 1 and 2 integrons in *Shigella flexneri *and *Shigella sonnei *isolated in Uzbekistan

**DOI:** 10.1186/1757-4749-2-18

**Published:** 2010-12-09

**Authors:** Ruslan S Madiyarov, Amir M Bektemirov, Gulnara A Ibadova, Gulnara K Abdukhalilova, Aybek V Khodiev, Ladaporn Bodhidatta, Orntipa Sethabutr, Carl J Mason

**Affiliations:** 1 Research Institute of Epidemiology, Microbiology and Infectious Diseases, Tashkent 100133, Republic of Uzbekistan; 2 Department of Enteric Diseases, Armed Forces Research Institute of Medical Sciences (AFRIMS), 315/6 Rajvithi Road, Bangkok 10400, Thailand

## Abstract

**Background:**

Shigella is a frequent cause of bacterial dysentery in the developing world. Treatment with effective antibiotics is recommended for shigellosis, but options become limited due to globally emerging resistance. One of the mechanisms for the development of resistance utilizes integrons. This study described the antibiotic susceptibility and the presence of class 1 and 2 integrons in *S. flexneri *and *S. sonnei *isolated in Uzbekistan.

**Results:**

We studied 31 isolates of *S. flexneri *and 21 isolates of *S. sonnei *isolated in Uzbekistan between 1992 and 2007 for the susceptibility or resistance to ampicillin (Am), chloramphenicol (Cl), tetracycline (Te), co-trimoxazole (Sxt), kanamycin (Km), streptomycin (Str), gentamicin (Gm), cefazolin (Czn), cefoperazone (Cpr), cefuroxime (Cur), ceftazidime (Ctz), nalidixic acid (NA) and ciprofloxacin (Cip). Am/Str/Cl/Te and Am/Str/Cl/Te/Sxt resistance patterns were found most frequently in *S. flexneri*. Single isolates were resistant to aminoglycoside, quinolones and cephalosporins. The resistance patterns were different in the two species. Integrons were detected in 93.5% of *S. flexneri *(29/31) and 81.0% of *S. sonnei *(17/21) isolates. In addition, 61.3% of *S. flexneri *(19/31) isolates and 19.0% of *S. sonnei *(4/21) isolates carried both classes of integrons. In 29.0% of *S. flexneri *(9/31) isolates, only class 1 integrons were identified. In *S. flexneri *isolates, the presence of class 1 integrons was associated with resistance to ampicillin and chloramphenicol. Only Class 2 integrons were present in 61.9% of *S. sonnei *(13/21) isolates.

**Conclusions:**

Our study documents antibiotic resistance among *Shigella *spp. in Uzbekistan. Ninety percent of *Shigella *strains were resistant to previously used antibiotics. Differences among *S. flexneri *and *S. sonnei *isolates in patterns of antimicrobial resistance to routinely used shigellosis antibiotics were observed. The majority of *S. flexneri *were resistant to ampicillin, chloramphenicol, tetracycline and streptomycin. Class 1 and 2 integrons were widely present in these *Shigella *strains. Resistance to ampicillin/chloramphenicol was associated with the presence of class 1 integrons. Though several mechanisms are possible, the resistance of *Shigella *isolates to ampicillin/chloramphenicol may be associated with the expression of genes within class 1 integrons.

## Background

Shigellosis is an acute enteric infection caused by Shigella species and manifested by diarrhea. Shigellosis is endemic to many developing countries, and also occurs in epidemics causing considerable morbidity and mortality [1,2]. It was reported that more than 140 million cases of shigellosis occurred worldwide, with 600,000 people dying annually; 60% of the deaths were seen in children under the age of 5 [3]. *S. flexneri *is the most commonly isolated Shigella species in the developing world and the most frequent cause of bacterial dysentery. In countries with improved water supply and sanitation, the predominant species is *S. sonnei*. Antimicrobial agents are recommended for the treatment of shigellosis; however, increasing antimicrobial resistance in the *Shigella *spp. has been reported worldwide [1,2]. *Shigella *strains have obtained resistance to some of the most widely used antibiotics, resulting in reduced efficacies of antimicrobial therapy [2,4-6]. One of the mechanisms of resistance development is the horizontal transfer of genetic factors. These include R-plasmids, transposons, integrons and genetic "islands", which can transfer within a taxon [7,8]. Integrons, discovered in the 1980s, are able to horizontally transfer genes; this is common for *Enterobacteriaceae *[9,10]. Integrons typically consist of an *intI *gene, which encodes for integrase that catalyzes the incorporation or excision of gene cassettes by site-specific recombination; a recombination site *attI*; and a promoter responsible for the expression of inserted gene cassettes. Integrons have been extensively studied in clinical environments due to their association with other mobile genetic elements and multi-resistance phenotypes. There are 4 classes of integrons that are distinguishable by the integrase enzymes [11,12]. Class 1 and 2 integrons are the most widespread among the *Enterobacteriaceae*, and the correlation between the presence of integrons and resistance to some antimicrobial agents has been demonstrated [13]. There are several publications dedicated to the study of integrons in *Shigella *spp. in different regions; however, Central Asia was not covered in these studies [14-16]. The widespread prevalence of poly-resistant enteric bacteria necessitates the consistent surveillance for antimicrobial resistance in *Shigella *spp. to select effective antimicrobial agents. The purpose of this study was to analyze antimicrobial susceptibility and the presence of integrons in *S. flexneri *and *S. sonnei *isolated in Uzbekistan.

## Results and Discussion

We studied 31 isolates of *S. flexneri *and 21 isolates of *S. sonnei *that were isolated in Uzbekistan between 1992 and 2007 for the susceptibility or resistance to ampicillin (Am), chloramphenicol (Cl), tetracycline (Te), co-trimoxazole (Sxt), kanamycin (Km), streptomycin (Str), gentamicin (Gm), cefazolin (Czn), cefoperazone (Cpr), cefuroxime (Cur), ceftazidime (Ctz), nalidixic acid (NA) and ciprofloxacin (Cip). All Shigella isolates were resistant to streptomycin. Most of the isolates were resistant to ampicillin, chloramphenicol and tetracycline (Table 1). Among the *S. flexneri *isolates, the most prevalent antimicrobial agent resistance patterns were Am/Str/Cl/Te and Am/Str/Cl/Te/Sxt. The most common *S. sonnei *antimicrobial agent resistance patterns were Str, Str/Sxt/Te and Str/Te (Table 2).

**Table 1:** Antimicrobial agent susceptibilities and MIC ranges of Shigella isolates from Uzbekistan

Antimicrobial agent*	*S. flexneri *(n = 31)				*S. sonnei *(n = 21)				**Breakpoint values (μg/ml) **^**||**^		
				
	Resistant isolates	**MIC**^**† **^**(μg/ml)**			Resistant isolates	MIC (μg/ml)					
	No (%)	Range	**MIC**_**50**_^**‡**^	**MIC**_**90**_^**§**^	No (%)	Range	**MIC**_**50**_	**MIC**_**90**_	R	I	S
Am	28(90.4)	2-512	256	512	1(4.8)	4-512	4	8	≥32	16	≤8
Cl	28(90.4)	4-256	128	256	1(4.8)	2-256	8	8	≥32	16	≤8
Te	29(93.5)	1-256	128	128	7(66.7)	1-256	128	256	≥16	8	≤4
Sxt	10(32.3)	0.5-128	2/38	128/2432	6(62)	0.5-128	128/2432	128/2432	≥4/76	N/A	≤2/38
Km	2(6.5)	4-128	8	16	1(4.8)	4-64	8	8	≥64	32	≤16
Str	31(100)	64-256	256	256	21(100)	256	256	256	≥64	32	≤16
Gm	1(3.2)	2-32	4	4	2(9.5)	1-64	2	2	≥16	8	≤4
Czn	1(3.2)	0.5-32	2	4	0(0.0)	1-4	2	2	≥32	16	≤8
Cpr	1(3.2)	0.03-64	0.25	0.5	0(0.0)	0.12-0.5	0.12	0.25	≥64	32	≤16
Cur	1(3.2)	1-64	4	4	0(0.0)	1-4	4	4	≥32	16	≤8
Ctz	1(3.2)	0.06-64	0.12	0.25	0(0.0)	0.06-0.5	0.12	0.25	≥32	16	≤8
NA	0(0.0)	0.12-0.5	0.12	0.5	1(4.8)	0.12-32	0.12	0.25	≥32	N/A	≤16
Cip	0(0.0)	0.008-0.016	0.008	0.016	1(4.8)	0.004-4	0.008	0.016	≥4	2	≤1

* Am = ampicillin, Cl = chloramphenicol, Te = tetracycline, Sxt = co-trimoxazole (trimethoprim- sulfamethoxazole), Km = kanamycin, Str = streptomycin, Gm = gentamicin, Czn = cefazolin, Cpr = cefoperazone, Cur = cefuroxime, Ctz = ceftazidime, NA = nalidixic acid, Cip = ciprofloxacin

† MIC = Minimal inhibitory concentration; ‡ MIC_50 _= MIC inhibiting 50% of isolates; § MIC_90 _= MIC inhibiting 90% of isolates; || - intermediate resistant isolates were considered as resistant

**Table 2:** Antimicrobial agent resistance patterns of *S. flexneri* and *S. sonnei* isolates from Uzbekistan

Antimicrobial resistance pattern*	*S. flexneri *(n = 31) No (%)	*S. sonnei *(n = 21) No (%)
Am/Cl/Str/Te	19 (61.3)	0 (0.0)
Am/Cl/Str/Sxt/Te	8 (25.8)	0 (0.0)
Am/Cl/Str/Sxt/Te/Czn/Cpr/Cur/Ctz/Gm/Km	1 (3.2)	0 (0.0)
Km/Str/Sxt	1 (3.2)	0 (0.0)
Str/Sxt	2 (6.4)	0 (0.0)
Str/Sxt/Te	0 (0.0)	10 (47.6)
Str	0 (0.0)	6 (28.6)
Str/Te	0 (0.0)	2 (9.5)
Am/Cl/Str/Sxt/Gm/Km	0 (0.0)	1 (4.8)
Gm/Str/Sxt/Te	0 (0.0)	1 (4.8)
NA/Cip/Str/Sxt/Te	0 (0.0)	1 (4.8)

* Am = ampicillin, Cl = chloramphenicol, Te = tetracycline, Sxt = co-trimoxazole (trimethoprim- sulfamethoxazole), Km = kanamycin, Str = streptomycin, Gm = gentamicin, Czn = cefazolin, Cpr = cefoperazone, Cur = cefuroxime, Ctz = ceftazidime, NA = nalidixic acid, Cip = ciprofloxacin

Our data revealed differences in antimicrobial agent resistance patterns among *S. flexneri *and *S. sonnei *for the routinely used antimicrobial agents. The *S. sonnei *isolates retained high sensitivity to ampicillin and chloramphenicol but also exhibited higher resistance to co-trimoxazole than the *S. flexneri *isolates did. However, we identified one *S. flexneri *isolate that resistant to all tested cephalosporins and one *S. sonnei *isolate resistant to nalidixic acid and the cephalosporins.

Ampicillin, chloramphenicol and tetracycline were the medications of choice in Uzbekistan for the treatment of shigellosis. Currently, 90% of clinical isolates of *S. flexneri*, the most common cause of shigellosis in Uzbekistan, have lost the natural susceptibility to these medications. In addition, the appearance of strains resistant to quinolones and cephalosporins, which are recommended for treatment, is an emerging problem that will require new approaches for antimicrobial therapy of shigellosis [17]. However, in our study, we observed high *in vitro *activity of quinolones and cephalosporins, making them the recommended drugs of choice for the treatment of shigellosis in Uzbekistan [2,18].

PCR studies for integrons revealed the presence of both class 1 and class 2 integrons in *S. flexneri *and *S. sonnei *(Tables 2 and 3). Integrons were detected in 93.5% of *S. flexneri *(29/31) and 81.0% of *S. sonnei *(17/21) isolates. In 61.3% of *S. flexneri *(19/31) isolates, both classes of integrons were identified, whereas only class 1 integrons were identified in 29.0% of *S. flexneri *(9/31) isolates. Within the *S. sonnei *isolates, class 2 integrons alone were identified in 61.9% of isolates (13/21) and both class 1 and 2 integrons were identified in 19.0% of isolates (4/21). The prevalence of class 2 integrons in *S. sonnei *is in agreement with other published data [16,19]. We did not identify *S. sonnei *isolates harboring only class 1 integrons.

**Table 3:** Presence of integrons in Shigella isolates from Uzbekistan and sensitivity/resistance to selected antimicrobial agents

**Isolate no**.	Species	Antimicrobial agent*							Integron class	
		
		Am	Cl	Gm	Km	Sxt	Te	Str	1	2
1410	*S. flexneri *1a	R	R	S	S	S	R	R	+	-
1806	*S. flexneri *1b	R	R	S	S	S	R	R	+	+
2316	*S. flexneri *1c	R	R	S	S	S	R	R	+	-
1461/542	*S. flexneri *2a	R	R	S	S	S	R	R	+	-
2125/947	*S. flexneri *2a	R	R	S	S	S	R	R	+	-
2864/574	*S. flexneri *2a	R	R	S	S	R	R	R	+	+
3104/355	*S. flexneri *2a	R	R	S	S	R	R	R	+	+
3116/944	*S. flexneri *2a	R	R	S	S	R	R	R	+	+
3160/383	*S. flexneri *2a	R	R	S	S	R	R	R	+	-
4036/750	*S. flexneri *2a	R	R	S	S	S	R	R	+	+
33	*S. flexneri *2a	R	R	S	S	S	R	R	+	+
430	*S. flexneri *2a	R	R	S	S	S	R	R	+	-
525	*S. flexneri *2a	R	R	S	S	R	R	R	+	+
842	*S. flexneri *2a	R	R	S	S	R	R	R	+	+
1773	*S. flexneri *2a	R	R	S	S	S	R	R	+	+
1771	*S. flexneri *2a	R	R	S	S	S	R	R	+	+
1807	*S. flexneri *2a	R	R	S	S	S	R	R	+	+
1832	*S. flexneri *2a	R	R	S	S	R	R	R	+	+
2011	*S. flexneri *2a	R	R	S	S	S	R	R	+	+
2344	*S. flexneri *2a	R	R	S	S	S	R	R	+	+
2215/278	*S. flexneri *2a	S	S	S	R	R	S	R	-	+
737	*S. flexneri *2a	S	S	S	S	S	R	R	-	-
1875	*S. flexneri *2b	R	R	S	S	R	R	R	+	+
4297/499	*S. flexneri *2b	R	R	S	S	S	R	R	+	+
1874	*S. flexneri *3a	R	R	S	S	S	R	R	+	+
769	*S. flexneri *3b	R	R	S	S	S	R	R	+	-
1544	*S. flexneri *4	R	R	R	R	R	R	R	+	+
2376	*S. flexneri *4	R	R	S	S	S	R	R	+	-
3049/4396	*S. flexneri *6	R	R	S	S	S	R	R	+	+
2078	*S. flexneri *6	R	R	S	S	S	R	R	+	-
2126/4720	*S. flexneri *6	S	S	S	S	S	S	R	-	-
344/1899	*S. sonnei*	S	S	S	S	S	S	R	-	-
346/459	*S. sonnei*	S	S	S	S	R	R	R	+	+
358/477	*S. sonnei*	S	S	S	S	S	S	R	-	+
333/1755	*S. sonnei*	S	S	S	S	S	R	R	+	+
359/400	*S. sonnei*	S	S	S	S	S	S	R	-	+
353/1950	*S. sonnei*	S	S	S	S	R	R	R	-	+
766/601	*S. sonnei*	S	S	S	S	S	R	R	-	-
1159/107	*S. sonnei*	S	S	S	S	R	R	R	-	-
1201/1982	*S. sonnei*	S	S	S	S	R	R	R	-	+
1316/152	*S. sonnei*	S	S	S	S	S	S	R	-	+
1805/146	*S. sonnei*	S	S	S	S	R	R	R	+	+
1849/760	*S. sonnei*	S	S	S	S	S	S	R	-	+
2939/5116	*S. sonnei*	S	S	S	S	S	S	R	-	-
1553/3	*S. sonnei*	R	R	R	R	R	S	R	+	+
1711/2	*S. sonnei*	S	S	S	S	R	R	R	-	+
1745/3	*S. sonnei*	S	S	S	S	R	R	R	-	+
4166/149	*S. sonnei*	S	S	S	S	R	R	R	-	+
4336/671	*S. sonnei*	S	S	S	S	R	R	R	-	+
4340/966	*S. sonnei*	S	S	S	S	R	R	R	-	+
4342/1199	*S. sonnei*	S	S	S	S	R	R	R	-	+
4345/199	*S. sonnei*	S	S	R	S	R	R	R	-	+

* S = susceptible, R = resistant, * Am = ampicillin, Cl = chloramphenicol, Te = tetracycline, Sxt = co-trimoxazole (trimethoprim- sulfamethoxazole), Km = kanamycin, Str = streptomycin, Gm = gentamicin, Czn = cefazolin, Cpr = cefoperazone, Cur = cefuroxime, Ctz = ceftazidime, NA = nalidixic acid, Cip = ciprofloxacin

The high prevalence of class 1 and class 2 integrons in this series of *Shigella *isolates as well as high prevalence of resistance to the "older" antibiotics: ampicillin, chloramphenicol, tetracycline, and streptomycin, makes statistical associations between phenotypic antimicrobial resistance and the presence of integrons difficult to interpret. In this series of isolates, for example, resistance to streptomycin was detected in all 52 *Shigella *isolates yet two *S. flexneri *and four *S. sonnei *isolates had no integrons detected (Table 3). Yet, in all 28 *S. flexneri *isolates where class 1 integrons were detected, resistance to both chloramphenicol and ampicillin was noted, and those 3 *S. flexneri *isolates lacking class 1 integrons were susceptible to both. Although cassettes including resistance genes to ampicillin, chloramphenicol, and streptomycin have all been reported to commonly be present in integrons, in the absence of sequencing augmented by conjugation experiments, it is not possible to confirm the location of the active resistance genes in integrons [11,15,18].

## Conclusions

Our study confirms the circulation of *Shigella flexneri *and *Shigella sonnei *strains that are resistant to widely used antimicrobial agents and significant prevalence of integrons in *Shigella *spp. circulating in Uzbekistan. Though several mechanisms are possible, the resistance of *Shigella *isolates to ampicillin/chloramphenicol may be associated with the expression of genes within class 1 integrons. Cephalosporins and quinolones retain high *in vitro *activity, but emergence of strains resistant to all tested cephalosporins and other antimicrobials will require continued routine monitoring of the antimicrobial resistance of *Shigella *spp. in Uzbekistan.

## Methods

### Isolates and serotyping

All Shigella isolates were provided by the "National Collection of Pathogens I-II Group" of the Ministry of Health of the Republic of Uzbekistan. In total, 52 isolates of *S. flexneri *and *S. sonnei *were studied (31 and 21 isolates, respectively). All strains were isolated from stool samples of patients with bacterial dysentery in Uzbekistan between 1992 and 2007. The isolation and confirmation of Shigella species were performed by following WHO recommendations [20]. Stool samples were inoculated on Endo agar and Salmonella-Shigella agar (SS agar) (Merck, Darmstadt, Germany). After overnight incubation at 37°C, the Endo agar and SS agar plates were checked for non-lactose-fermenting colonies. Colonies characteristically resembling *Shigella *spp. were differentiated from other non-lactose-fermenting enteric pathogens by inoculating on Kligler iron agar (KIA agar) (HiMedia Laboratories Pvt. Ltd., Mumbai, India) for typical reactions [20]. Biochemical and serological typing of *Shigella *spp. were performed by standard methods with specific antisera [21]. Prior to testing, each isolate was grown on a Mueller-Hinton agar (MHA) plate (HiMedia Laboratories Pvt. Ltd., Mumbai, India), incubated overnight at 37°C and subsequently checked for auto-agglutination in saline. Specimens grown on the 18-20 hrs agar plate were used to carry out agglutination tests according to the manufacturers' instructions (SPBNIIVS, Sankt-Petersburg, Russian Federation). In cases where no agglutination was observed with any of the commercial antisera, the isolates were tested for the presence of heat-labile inhibitory substances as recommended [21]. Briefly, a colony was picked with loop and dissolved in sterile normal saline to 0.5 McFarland standards, heated at 100°C for 60 min, cooled and centrifuged; and resulting pellets were used for testing. For the slide agglutination test, the agglutination of prepared samples with antisera was observed against a black background. The slide was tilted for 1 min and agglutination scores were graded from 0 to 4 (0-no agglutination after 1 min, 1-weak agglutination after 3 min, 2-weak agglutination after 1 min, 3-agglutination within 1 min, 4-visible rapid agglutination right after addition of antisera). Only 3-4 scored agglutinated samples were considered as positive.

### Antimicrobial susceptibility test

Antimicrobial susceptibility testing for Minimal Inhibitory Concentration (MIC50 and MIC90) was performed by a serial dilution method on solid agar, and the Minimal Inhibitory Concentration was determined on MHA according to Clinical and Laboratory Standards Institute and the "Guidelines for susceptibility testing of microorganisms to antimicrobial agents" (Ministry of Health, Russian Federation) [22-24].

Briefly, MHA plates with different concentrations of antimicrobial agents ((HiMedia Laboratories Pvt. Ltd., Mumbai, India) were prepared. Plates without antimicrobial agents were prepared for growth quality control. MHA was poured into 90-mm plates on 3 mm depth. Once prepared, plates were stored at 4°C and used within 2 days. Approximately four colonies of overnight incubated subcultures were inoculated into 4 ml of Nutrient broth (HiMedia Laboratories Pvt. Ltd., Mumbai, India). After 4 h of incubation at 37°C, bacterial suspension was adjusted to 0.5 McFarland standards. A 100 μl volume of this suspension was added to 900 μl of broth to produce a concentration of approximately 1 × 10^7 ^CFU/ml. Plates were inoculated with metal loop (approximately 1 μl of bacterial suspension resulting spot of 5 mm in diameter) with 1 × 10^4 ^CFU/ml in final inoculum. Plates were incubated for 18 h at 35°C under atmospheric conditions. Growth was recorded positive if at least one colony was observed at the inoculation site [22-24]. Isolates were identified as resistant or susceptible (R/S). Intermediate susceptibility was categorized as resistant. Reference strains of *E. coli *(ATCC 25922), *S. aureus *(ATCC 25923) and *P. aeruginosa *(ATCC 27853) were used for media and reagent quality control.

### Integron detection

Bacterial DNA was extracted from overnight incubation of pure cultures by heating in a water bath at 96°C for 20 min. After centrifugation, supernatants were stored at -20°C prior to PCR reactions. Primers (Operon Biotechnologies GmbH, Cologne, Germany) int1L (5'-ACATGTGATGGCGACGCACGA-3') and int1R (5'-ATTTCTGTCCTGGCTGGCGA-3') were used for amplification of class 1 integrase (initial denaturation at 94°C for 10 min; 35 cycles of 94°C for 1 min, 60°C for 1 min, 72°C for 1 min; final elongation at 72°C for 10 min) [18]; Primers int2F (5'-GTAGCAAACGAGTGACGAAATG-3') and int2R (5'-CACGGATATGCGACAAAAAGGT-3') were used for amplification of class 2 integrase (initial denaturation at 95°C for 10 min; 35 cycles of 95°C for 50 sec, 58°C for 1 min, 72°C for 1 min10 sec; final elongation at 72°C for 10 min) [25]. The amplification was performed with the FailSafe™PCR System (EPICENTRE Biotechnologies, Madison, WI, USA) in a 25.0 μl reaction volume on a Thermo Px2 thermal cycler (Thermo Fisher Scientific Inc., Waltham, MA, USA) following manufacturer recommendations. PCR products were detected by ethidium bromine staining after electrophoresis in 1.5% agarose gels (SERVA Electrophoresis GmbH, Heidelberg, Germany), with DNA ladders (pUC19DNA/Mspl-Silex, Moscow, Russian Federation; M100-Galart-diagnosticum, Moscow, Russian Federation) to determine band sizes. The expected band sizes were approximately 569 bp and 789 bp for the class 1 and class 2 integrase PCR products [18,25].

## Competing interests

The authors declare that they have no competing interests.

## Authors' contributions

All authors equally participated in study design, data analysis/interpretation and writing of the manuscript.
